# Sequential Measurements of Pentraxin 3 Serum Levels in Patients with Ventilator-Associated Pneumonia: A Nested Case-Control Study

**DOI:** 10.1155/2018/4074169

**Published:** 2018-05-13

**Authors:** Huseyin Bilgin, Murat Haliloglu, Ali Yaman, Pinar Ay, Beliz Bilgili, Mustafa Kemal Arslantas, Filiz Ture Ozdemir, Goncagul Haklar, Ismail Cinel, Lutfiye Mulazimoglu

**Affiliations:** ^1^Infectious Diseases and Clinical Microbiology, Marmara University School of Medicine, Istanbul, Turkey; ^2^Department of Anesthesiology and Intensive Care, Marmara University School of Medicine, Istanbul, Turkey; ^3^Department of Biochemistry, Marmara University School of Medicine, Istanbul, Turkey; ^4^Department of Public Health, Marmara University School of Medicine, Istanbul, Turkey; ^5^Department of Immunology, Marmara University School of Medicine, Istanbul, Turkey

## Abstract

**Purpose:**

The main purpose of this study was to investigate the dynamics of pentraxin 3 (PTX3) compared with procalcitonin (PCT) and C-reactive protein (CRP) in patients with suspicion of ventilator-associated pneumonia (VAP).

**Materials and Methods:**

We designed a nested case-control study. This study was performed in the Surgical Intensive Care Unit of a tertiary care academic university and teaching hospital. Ninety-one adults who were mechanically ventilated for >48 hours were enrolled in the study. VAP diagnosis was established among 28 patients following the 2005 ATS/IDSA guidelines.

**Results:**

The median PTX3 plasma level was 2.66 ng/mL in VAP adults compared to 0.25 ng/mL in non-VAP adults (*p* < 0.05). Procalcitonin and CRP levels did not significantly differ. Pentraxin 3, with a 2.56 ng/mL breakpoint, had 85% sensitivity, 86% specificity, 75% positive predictive value, and 92.9% negative predictive value for VAP diagnosis (AUC = 0.78).

**Conclusions:**

With the suspicion of VAP, a pentraxin 3 plasma breakpoint of 2.56 ng/mL could contribute to the decision of whether to start antibiotics.

## 1. Introduction

For patients with suspected ventilator-associated pneumonia (VAP), the 2016 ATS/IDSA guidelines [1] recommend using clinical criteria alone, rather than combining those criteria with procalcitonin (PCT) or C-reactive protein (CRP) to decide whether to start antibiotics. Besides, interobserver variability in interpreting subjective criteria, such as chest X-rays and respiratory secretions, is problematic [2, 3], making the diagnosis of VAP controversial. Indeed, VAP is responsible for 50% of antibiotic consumption in critically ill patients [4, 5]. The lack of objectivity and of a consensus regarding clinical criteria causes overdiagnosis of VAP, excessive use of antibiotics, and difficulties in benchmarking [6]. Diagnosis of VAP is complicated by the potential overlap with tracheobronchitis [7].

Pentraxin 3 (PTX3) is an acute-phase inflammatory mediator whose levels increase rapidly in inflammatory and infectious conditions. Increased PTX3 levels are correlated with the severity of lung injury and infection [8, 9]. Thus, we hypothesized that plasma PTX3 levels may aid in VAP diagnosis. The purpose of this study was to investigate the potential role of pentraxin 3, PCT, and CRP in VAP diagnosis. A prospective nested case-control study was performed with sequential measurement of pentraxin 3, procalcitonin, and CRP levels in patients receiving mechanical ventilation in the Surgical Intensive Care Unit, with the aim of determining the utility of biomarker levels in the diagnosis of VAP and establishing a cutoff point.

## 2. Methods

### 2.1. Study Population

We designed a prospective nested case-control study in the Surgical Intensive Care Unit (ICU) of a 650-bed tertiary teaching university hospital conducted between July 2015 and June 2016. The study protocol was approved by the local ethics committee of Marmara University School of Medicine (reference number: 09.2015.134). Informed consent forms were obtained from patients or their legally authorized representative.

**Figure 1:**
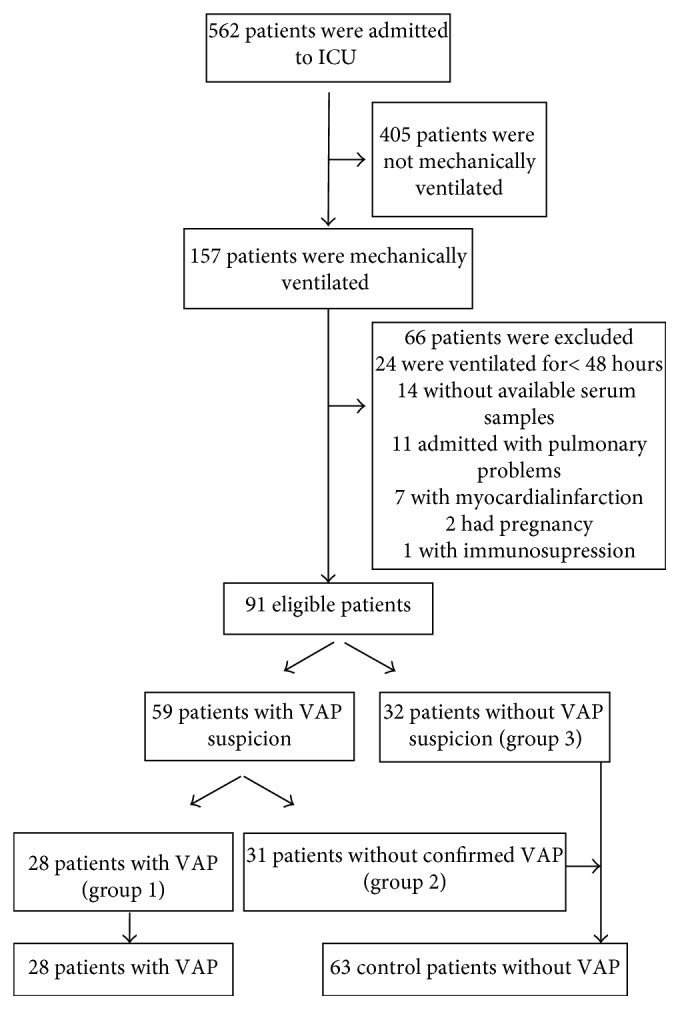
Flow of the study.

Patients who underwent mechanical ventilation (MV) > 48 hours and who were >17 years of age were screened daily, and serum samples of all patients were collected. We excluded patients whose serum samples were not available and patients who were admitted to the ICU with pneumonia, pulmonary emboli, myocardial infarction, pregnancy, or lung cancer, considering the elevated PTX3 levels found in these cases. Patients with MV < 48 hours were also excluded (Figure 1). Biomarker level measurements were not waited for the decision to start antibiotics for VAP.

### 2.2. Variables and Measures

Patients were followed up daily with prospective chart review and communication with attending physicians. Basic demographics, comorbidities, reasons for admission, lengths of stay in the ICU and in the hospital, surgical procedures, and antibiotic usage data prior to inclusion were recorded using a predesigned data collection form. Daily evaluations for the clinical diagnostic criteria of VAP (temperature, leukocytes, oxygenation, tracheal secretions, and pulmonary radiography) were performed. Antimicrobial usage data on the day of respiratory sample collection were collected.

Acute Physiology and Chronic Health Evaluation (APACHE) II scores were calculated on the day of admission [10]. Sequential Organ Failure Assessment (SOFA) scores, Clinical Pulmonary Infection Score (CPIS), and PaO_2_/FiO_2_ ratios were calculated at the beginning of MV and in the days thereafter [11, 12]. Patients were followed up until the 28th day (D28) after the suspicion of VAP or mechanical ventilation.

### 2.3. Definitions

Patients were defined as confirmed VAP patients (group 1), clinically suspected VAP patients (group 2), and non-VAP controls (group 3) by prospective follow-up. Suspicion of VAP was established using the classical clinical criteria: a new and persistent radiographic infiltrate together with two of the following criteria: temperature of >38°C or <36°C, leukocytes > 12 × 10^3^ or <4 × 10^3^ mm^3^, or purulent tracheal aspirate [13]. Confirmation of VAP was defined as clinically suspected VAP plus positive microbiological result. Quantitative culture of ≥1 × 10^5^ cfu/mL in endotracheal secretion samples was recorded as a positive microbiological result. The patients who were suspected as VAP according to the clinical criteria without positive microbiological test were allocated to non-VAP control patients. D0 was defined as the beginning of MV, and Dv was defined as VAP suspicion day.

The diagnosis team consisting of two expert intensive care unit and one infectious disease doctors evaluated the clinical and microbiologic information, blinded to the biomarker results, and assigned the final diagnosis of VAP. The final decision on discontinuation of antibiotic therapy was left to the attending physician.

### 2.4. Data Collection

Group of controls was formed by matching patients with non-VAP patients. Non-VAP patients were matched according to previous duration of mechanical ventilation until the infection, equal to previous duration of ventilation in VAP case minus one and plus one day; if previous ventilation duration is longer than 14 days in the VAP case, then minus one and up to minus three days. Biomarker levels of VAP patients on the day of VAP diagnosis were compared with the biomarker levels on the matched ventilation day of control patients.

Blood samples for the determination of CRP, PCT, and PTX3 were collected on D0, D1, D2, D5, D7, D10, and D14. Samples were frozen at −80°C after centrifugation. Measurements were performed in the central laboratory of Marmara University Pendik Hospital. CRP and PCT levels (Brahms PCT Elecsys, Roche) were determined by an immunoturbidimetric method using a commercially available kit. The levels of PTX3 were measured using a commercial solid-phase enzyme-linked immunosorbent assay (ELISA) according to the manufacturer's instructions (Boster Biological Technology Co., Ltd.). The plates were read at a wavelength of 450 nm using an automatic ELISA reader. The detection limit was 0.1 ng/mL, and the assay did not cross-react with CRP or serum amyloid A protein.

### 2.5. Statistical Analysis

Sample size analysis was performed using MedCalc, version 15.2 (MedCalc Software, Ostend, Belgium). We planned to include 96 patients (32 VAP and 64 controls) for a power of 90% (type II error = 0.10, type I error = 0.05, and ROC AUC = 0.700). To estimate the incidence of VAP, we analyzed the previous year's VAP rates. Between 1 July 2014 and 30 June 2014, 698 patients were admitted to SICU, and 45 patients were diagnosed with VAP.

Descriptive variables were defined using frequencies, percentages, means, standard deviations, medians, and percentiles. Categorical variables were compared using chi-square and Fisher's analyses when needed. Continuous variables with normal distribution were compared using *t*-tests, while variables with skewed distributions were compared using Mann–Whitney *U* tests. The sensitivity, specificity, and positive and negative predictive values of pentraxin 3, procalcitonin, and CRP levels were determined by comparing patients with and without VAP. The discriminatory ability of biomarkers was analyzed using the area under the receiver operating characteristic (ROC) curve. The sensitivity, specificity, positive predictive value (PPV), negative predictive value (NPV), and Youden's J index values were computed [14]. Optimal cutoff values for PTX3, PCT, and CRP were determined. The optimal cutoff values were obtained from the best sensitivity/specificity ratios. *p* < 0.05 was considered the level of statistical significance.

## 3. Results

### 3.1. Study Population

We enrolled 157 patients between July 2015 and March 2016, 66 of whom met the exclusion criteria. Among the 91 eligible patients, 59 were with suspicion of VAP and 32 were without suspicion of VAP (group 3). Among 59 patients, 28 of them had confirmed VAP (group 1) and 31 of them had clinically suspected VAP (group 2). 31 patients in group 2 were included in the control group along with the 32 patients without VAP suspicion (group 3) (Figure 1). The main characteristics of the patients are listed in Table 1. Endotracheal quantitative cultures were 1 × 10^4^ cfu/mL positive in four patients in group 2. Two of the patients in this group were on antibiotics during respiratory sample collection. Antimicrobial usage during respiratory sample collection did not differ between groups 1, 2, and 3. Also, CPISs were significantly higher in group 1 when compared to groups 2 and 3 (Table 1).

The median APACHE II and SOFA scores at the time of admission were similar between the two groups. The top three pathogens were *Acinetobacter baumannii*, Enterobacteriaceae, and *P. aeruginosa*. Median time to VAP diagnosis was 5 days (25–75% = 4–7.75 days). Mean antibiotic duration was 10.8 ± 1.9 days. Among 28 confirmed VAP patients, 9 of them were treated with piperacillin-tazobactam combination, 7 with carbapenem and colistin combination, 6 with 3rd or 4th generation cephalosporins, 4 with carbapenem monotherapy, and 1 with ampicillin-sulbactam combination. The median duration from VAP diagnosis to clinical response was 7 days (25–75% = 5–7 days).

### 3.2. VAP Prediction

**Figure 2:**
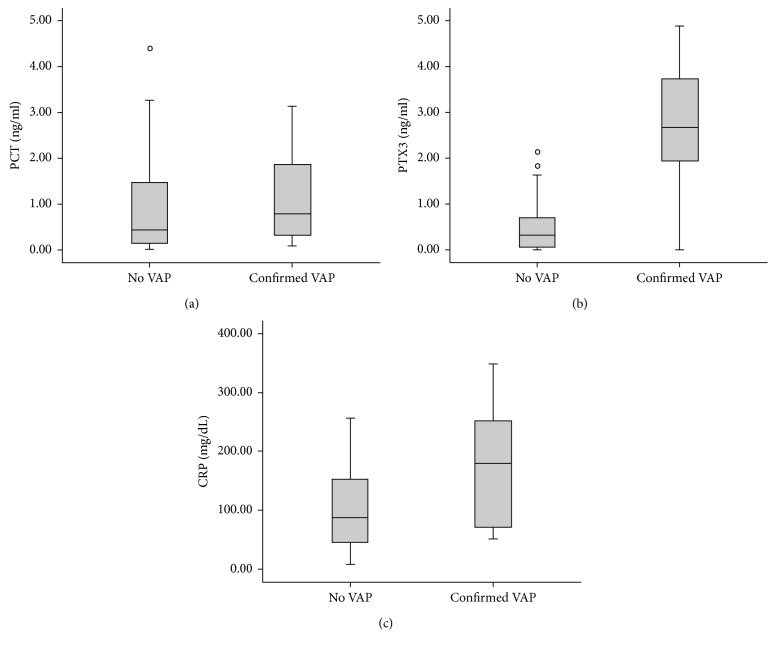
Levels of (a) PCT, (b) PTX3, and (c) CRP in confirmed VAP and non-VAP patients.

**Figure 3:**
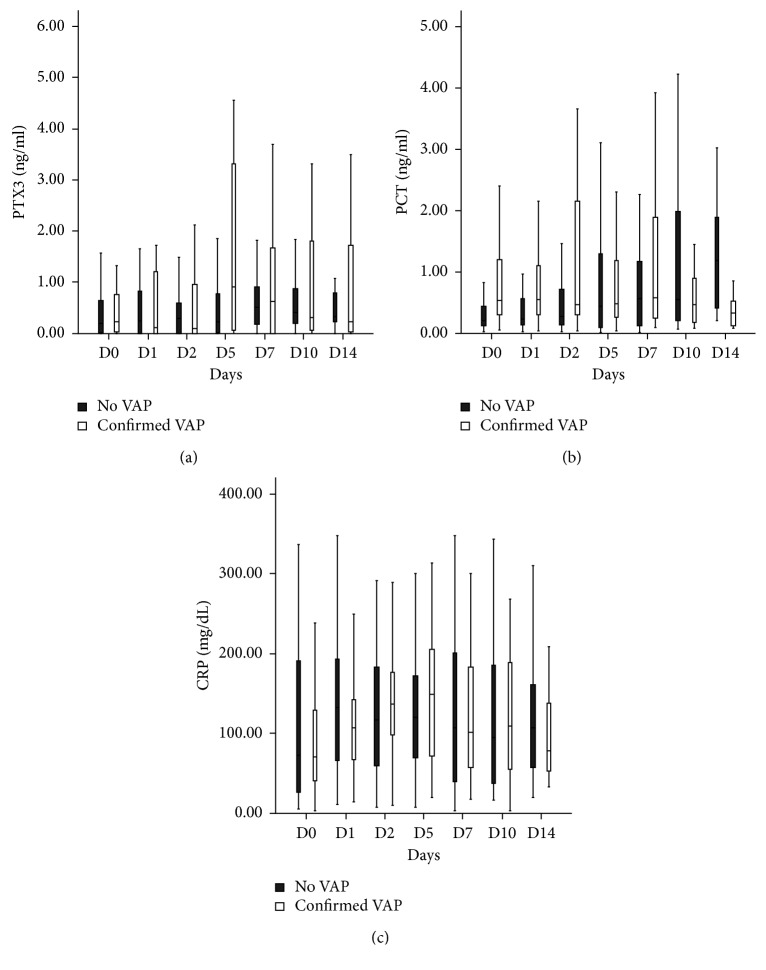
Sequential measurement levels of (a) PTX3, (b) PCT, and (c) CRP in VAP and non-VAP patients.

There was no significant difference in PTX3, PCT, and CRP levels between VAP and non-VAP patients on D0 (Table 1). Serum PTX3, PCT, and CRP levels were compared between Dv of the VAP group and matched mechanical ventilation days of the non-VAP group. The median PTX3 levels were significantly higher in the VAP group (2.66 ng/mL versus 0.25, *p* < 0.001). The levels of other biomarkers did not differ between the two groups (Figure 2). Sequential measurement levels of biomarkers are presented in Figure 3.

**Figure 4:**
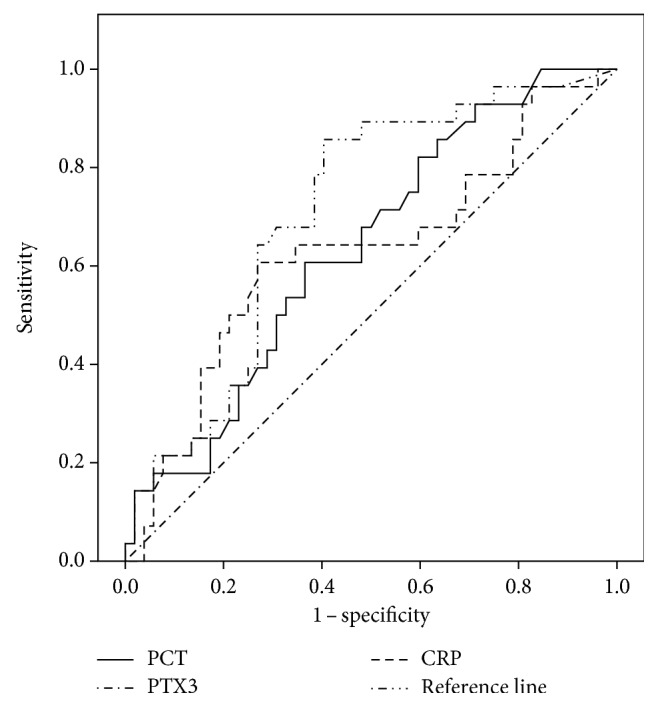
Receiver operative characteristic curve for pentraxin 3, procalcitonin, and C-reactive protein in ventilator-associated pneumonia diagnosis.

The ROC AUC analysis was performed for VAP diagnosis. Serum PTX3, PCT, and CRP levels were compared between Dv of the VAP group and matched mechanical ventilation days of the non-VAP group. The AUC was 0.78 (95% CI = 0.68–0.87) for PTX3, 0.581 for PCT (95% CI = 0.43–0.73), and 0.64 for CRP (95% CI = 0.49–0.79) (Figure 4). The optimal breakpoint of 2.56 ng/mL of PTX3 (identified by Youden's J index) was associated with 85% sensitivity, 86% specificity, 75% PPV, and 92.9% NPV. The total accuracy of PTX3 was 86.5%. For a PCT breakpoint of 1.45 ng/mL, sensitivity was 57%; specificity, 66%; PPV, 41%; and NPV, 75%. C-reactive protein had a sensitivity of 60%, a specificity of 80%, a PPV of 58%, and an NPV of 81% when using a breakpoint of 174 *μ*mol/L (Table 2).

Combination of pentraxin 3 > 2.56 ng/mL and procalcitonin > 1.45 ng/mL did not improve the sensitivity (46.4%) but increased the specificity (93.4%) for VAP diagnosis. Combination of pentraxin 3 > 2.56 ng/mL and CRP levels > 174 *µ*mol/L lowered the sensitivity to 53.6% but increased the specificity to 96.7%.

## 4. Discussion

The diagnosis of VAP is established by clinical criteria together with a positive quantitative culture of the respiratory sample.

Antibiotics for VAP are given for at least 72 hours until the culture results are determined. The overuse of antibiotics is common since the clinical criteria for VAP are still subjective [15]. Accurate and timely diagnosis is crucial regarding patient safety, antibiotic usage, resistance, and cost.

However, clinical diagnostic criteria for VAP remain questionable [3, 16]. Concerning microbiological confirmation, obtaining cultures is time-consuming, cutoff values for colony-forming units are variable, and the effect of the previous antibiotic causes problems and delay in the diagnosis of VAP. Thus, there is a need for rapid, accurate, and inexpensive diagnostic and prognostic methods for VAP [17–19].

This study indicates that PTX3 has a better performance than other biomarkers for VAP. Patients under the diagnostic breakpoint have a low probability of presenting with VAP. Our findings suggest that PTX3 may aid the decision to start antibiotics in patients with a suspicion of VAP. Combination of biomarkers did not improve the diagnostic capability.

Pentraxin 3 is elevated earlier than CRP in acute lung injury. Pentraxin 3 levels are correlated with acute lung injury, acute respiratory distress syndrome severity, and systemic involvement [20]. Several studies investigated the serum, pleural effusion, and alveolar levels of PTX3 in patients with pneumonia [9, 20, 21].

Pentraxin 3, in combination with clinical information, could be used to diagnose VAP in clinical practice. However, given the limitation of procalcitonin and CRP in VAP diagnosis, clinicians should not rely solely on biomarkers instead on clinical assessment.

A single-center study on VAP patients showed a diagnostic breakpoint level of 16.43 ng/mL, and sensitivity and specificity values were calculated as 74% and 68.6%, respectively. When compared to CRP, PTX3 was not superior in diagnosing VAP. Kao et al. reported that PTX3 levels could be used in the diagnosis and management of community-acquired pneumonia [22].

Mauri et al. investigated the role of bronchoalveolar lavage (BAL) and serum PTX3 levels along with other biomarkers in patients with VAP. Infected patients had significantly higher PTX3 BAL levels [20].

Our findings are consistent with previous studies and suggest that normal serum PTX 3 levels could be used to rule out VAP given its high negative predictive value.

Our results showed a low sensitivity and specificity for CRP and PCT in VAP diagnosis. CRP and PCT are used as parameters to monitor the clinical response in VAP [17, 18]. Recent VAP guidelines suggest that using PCT along with clinical criteria is required to monitor the treatment response and discontinue antibiotics [1].

There are several limitations to our study. First, this is a noninterventional study with a small sample size representing the results from a single center. Second, pentraxin 3 cutoff levels for VAP diagnosis are discordant with those reported in the literature [9]. We can explain this result with the use of a different ELISA kit and a difference in patient distribution. Discrimination ability of PTX3 measured by ROC curves is essential, but the absence of the gold standard for VAP diagnosis limits its usefulness. Finally, our findings were observed in a case mix of predominantly surgical and trauma patients with gram-negative infections. No validation cohort was used due to small sample size.

One of the strengths of the current study is that PTX3, CRP, and PCT were measured sequentially. Secondly, VAP and non-VAP patients were matched by age, sex, and mechanical ventilation day when comparing the biomarker levels.

## 5. Conclusion

The diagnostic performance of PTX3 was significantly superior to that of PCT and CRP. The measured level of serum PTX3 might be a reliable marker in the antibiotic decision-making process in patients with suspected VAP.

## Figures and Tables

**Table 1:** Baseline characteristics of the study patients.

Characteristics	Confirmed VAP (*n*=28)	Clinically suspected VAP (*n*=31)	Without VAP suspicion (*n*=32)	*p* value
Antimicrobial use during respiratory sample collection	12	11	17	0.36
Quantitative culture negative	0	27	32	0.01
Quantitative culture <1 × 10^5^ cfu/mL	0	4	0	0.01
Quantitative culture ≥1 × 10^5^ cfu/mL	28	0	0	0.01
CPIS, median (25–75%)	7 (3–7)	3 (1–5)	2 (1–3.7)	<0.01

*Characteristics*	*VAP,n*=28*(%)*	*Non-VAP controls,n*=63*(%)*		*pvalue*
Age (years), mean ± SD	54.2 ± 3.6	59.3 ± 2.2		0.88
Sex: female, *n* (%)	6 (21.4)	9 (29)		0.56
Days in ICU, median (25–75%)	28 (12.5–42)	9 (7–16.5)		0.001
Duration of MV (days), median (25–75%)	28 (11.5–36)	9 (6–16.5)		0.001
*Admission diagnosis*			
Emergency surgery	9 (32.1)	15 (24.6)		0.73
Elective surgery	6 (21.4)	20 (32.8)		0.73
Trauma	6 (21.4)	6 (9.8)		0.73
Medical	7 (25)	20 (32.8)		0.73
APACHE II score, median (25–75%)	21 (15–24)	20 (15–23.5)		0.50
SOFA score, median (25–75%)	6 (4–10)	8 (6–11)		0.07
CPIS, median (25–75%)	6 (3–7)	2 (1–5)		0.001
Crude mortality, *n* (%)	15 (53.6)	40 (65.6)		0.34
28-day mortality, *n* (%)	13 (46.4)	24 (40)		0.25
D0 pentraxin 3 (ng/mL)	0.32 (0.03–1.2)	0.06 (0.02–0.39)		0.07
D0 procalcitonin (ng/mL)	1.0 (0.32–2.7)	0.34 (0.18–4.37)		0.32
D0 CRP (*µ*mol/L)	70 (38.7–149.7)	100 (47–204)		0.19

ICU: intensive care unit; MV: mechanical ventilation; APACHE II: Acute Physiology and Chronic Health Evaluation; SOFA: Sequential Organ Failure Assessment; CPIS: Clinical Pulmonary Infection Score.

**Table 2:** Diagnostic value of serum levels of pentraxin 3, procalcitonin, and C-reactive protein.

	Optimal cutoff	AUC	Sensitivity	Sensitivity	NPV	PPV
Pentraxin 3	2.56	0.78	85	86	92.9	75
Procalcitonin	1.45	0.581	57	66	75	41
CRP	174	0.64	60	80	81	58

CRP: C-reactive protein.

## Data Availability

The data used to support the findings of this study are available from the corresponding author upon request.

## Abbreviations

ATS: American Thoracic Society
APACHE II: Acute Physiology and Chronic Health Evaluation
AUC: Area under the curve
BAL: Bronchoalveolar lavage
CDC: Centers of Disease Control
CPIS: Clinical Pulmonary Infection Score
CRP: C-reactive protein
ELISA: Enzyme-linked immunosorbent assay
ICU: Intensive care unit
IDSA: Infectious Diseases Society of America
MV: Mechanical ventilation
NPV: Negative predictive value
PCT: Procalcitonin
PPV: Positive predictive value
PTX3: Pentraxin 3
ROC: Receiver operating characteristic
SOFA: Sequential Organ Failure Assessment
VAP: Ventilator-associated pneumonia.
